# Understanding the Technology Acceptance and Usability of a New Device for Hand Therapy: Qualitative Descriptive Study

**DOI:** 10.2196/42385

**Published:** 2022-11-10

**Authors:** Adriana M Rios Rincon, Christine Guptill, Juan David Guevara Salamanca, Yilina Liubaoerjijin, Mathieu Figeys, Geoff Gregson, Antonio Miguel-Cruz

**Affiliations:** 1 Department of Occupational Therapy University of Alberta Edmonton, AB Canada; 2 Department of Occupational Therapy Faculté des Sciences de la Santé Université d'Ottawa Ottawa, ON Canada; 3 Department Glenrose Rehabilitation Research, Innovation & Technology Glenrose Rehabilitation Hospital Edmonton, AB Canada

**Keywords:** usability, technology acceptance, hand therapy, rehabilitation, disability, medical device, limb disorder, chronic disorder, health care system

## Abstract

**Background:**

Upper extremity function plays a critical role in completing activities of daily living, employment, and participating in recreational activities. The FEPSim device is a medical device for hand and wrist rehabilitation that can be adjusted according to the patient’s requirements in rehabilitation. Furthermore, the FEPSim can be used to assess the patient’s strength and range of motion of the forearm, wrist, and hand. At present, the acceptance and usability of the FEPSim have not been tested in a clinical setting, with limited perspectives from rehabilitation-providing clinicians.

**Objective:**

This study aims to understand the factors related to the acceptance and usability of the FEPSim device. Upper limb disorders are prevalent across populations. The impact of upper limb disorders, both acute and chronic, puts a significant burden on the Canadian health care system.

**Methods:**

A qualitative descriptive study was conducted that involved face-to-face semistructured interviews with hand therapists from hand therapy services who used the FEPSim device. We used purposive sampling to recruit 10 participants over a period of 14 months. Semistructured interview questions (topic-guided) examined the technology acceptance and usability of the FEPSim device.

**Results:**

We found 6 factors to be critical aspects of the acceptance and usability of the FEPSim device. These factors were (1) useful for therapy, (2) effortlessness, (3) environmental conditions, (4) internal encouragement, (5) technological aesthetics, and (6) use.

**Conclusions:**

The FEPSim device was widely accepted by the therapists. The use of the FEPSim device is a feasible alternative for supporting hand therapy.

**Trial Registration:**

ISRCTN Registry ISRCTN13656014; https://www.isrctn.com/ISRCTN13656014

## Introduction

Upper extremity function plays a critical role in completing activities of daily living, employment, and participating in recreational activities [1]. Upper extremity functions can be detrimentally impacted by numerous disorders, thus having a deleterious impact on health and well-being [1-4].

Upper limb disorders are prevalent across populations [5,6]. There is significant variability surrounding the operational definition of upper limb disorders with differing underlying etiologies, as well as significant heterogeneity [7]. Therefore, a wide prevalence of estimates of upper limb disorders between 1.6% and 53% has been reported [7].

The impact of upper limb disorders, both acute and chronic, puts a significant burden on the Canadian health care system. One of the most prevalent etiologies resulting in upper limb impairment is arthritis; nearly 1 in 5 Canadians are living with arthritis, resulting in a yearly health care expenditure of CAD $6.4 billion [8,9]. Another common cause is cerebrovascular accidents, with an incidence rate of approximately 62,000 cases per year, costing the health care system US $3.6 billion annually [10]. Nearly 714,000 cases of injuries specific to the wrist and hand were reported during 1 year, as reported by the Canadian Community Health Survey [11]. Focusing further on wrist fractures, nearly 47,000 cases per year are reported in Canada, resulting in over CAD $100 million in acute care costs alone [12]. Finally, people with spinal cord injury may also experience impaired hand function; 86,000 Canadians are living with spinal cord injury, resulting in CAD $2.7 billion of health care costs per year [13]. Together, etiologies resulting in upper limb disorders impact millions of Canadians and are associated with an annual health care expenditure in the tens of billions of dollars in Canada alone.

With this immense prevalence and impact of upper limb disorders, rehabilitative approaches play a pivotal role in compensation for, or restoration of function after, these impairments. The increasing emphasis on rehabilitation technologies to promote activities of daily living while increasing therapy efficiency, accessibility, and practicality has resulted in various technological and robotic devices targeting upper limb therapy [14-17]. Indeed, rehabilitation strategies can improve upper limb function, although compliance and technology acceptance and adoption can pose significant challenges [15]. Additional factors associated with conventional therapy approaches such as resistance balls and putties can quickly lead to a lack of engagement from the patient, which ultimately impacts performance and compliance. Robotic rehabilitative technologies have begun to address this issue, although often at a logistical or financial cost [15,17].

Current devices specific to upper limb rehabilitation can be categorized into low-cost and portable, high-cost and portable, or high-cost and nonportable [18]. Although many low-cost devices exist as part of routine clinical care, to the authors’ knowledge, none are capable of allowing a therapist to obtain performance metrics such as forearm, wrist, and hand strength; range of motion; or dexterity during functional hand movements (eg, wrist pronation/supination and flexion/extension) and grasp patterns (eg, the lateral grip, which is the grasp pattern used when grasping and turning a key to open a door). 

The FEPSim (flexion, extension, pronation, and supination), developed by Karma Medical Products, is a medical device for hand and wrist rehabilitation that can be adjusted according to the patient’s requirements in rehabilitation. Furthermore, the FEPSim can be used to assess the patient’s strength and range of motion of the forearm, wrist, and hand. To measure range of motion, the device has features that allow the therapist to determine the degrees of movement of any given joint. The FEPSim also counts the number of repetitions of an exercise that the patient performs, which is an indicator of the patient’s endurance. The customizability of the FEPSim allows for adjustments during rehabilitation progression and targeted therapeutic goals. This is achieved by the ability to adjust the strength and dexterity that the patient requires during therapy. The adjustment of dexterity is achieved by using a variety of accessories for exercising different grasp patterns such as disk grasp, power grasp, spherical grasp, and lateral grip. 

Although rehabilitative devices may be efficacious, there are factors behind technology acceptance, usability, and compliance that may ultimately impact the final adoption of these technologies. For instance, in a recent randomized controlled trial investigating neurorehabilitation technology with occupational therapy–delivered hand rehabilitation compared with occupational therapy alone, over one-third of the participants in the neurotechnology and therapy group dropped out of the study due to noncompliance [19]. Rates of nonadherence to rehabilitation interventions as high as 50% have been reported [20]. This puts further stressors on the individual, the therapist, and an overburdened health care system [20]. Therapists are critical stakeholders in the adoption of rehabilitation technologies. To facilitate adoption by therapists, factors including perceived technological effectiveness (ie, usefulness because technology helps to achieve the therapeutic goals), therapeutic effort to implement the device, and patient acceptance are fundamental [21]. 

Together, the FEPSim’s available features allow rehabilitation practitioners to optimize therapy, make informed decisions, and conduct objective measurements of rehabilitative progress specific to the upper limbs. At present, the acceptance and usability of the FEPSim have not been tested in a clinical setting, with limited perspectives from rehabilitation-providing clinicians. Therefore, the purpose of this study was to understand what factors affect the technology acceptance and usability of the FEPSim device for hand therapy by therapists at 2 hospitals in Canada.

## Methods

### Design

This study is part of a comprehensive study that aimed to determine the clinical effectiveness of adding the FEPSim device to standard care for patients with injuries and clinical conditions of the forearm, wrist, and hand. A comprehensive study protocol can be found in [18]. For the qualitative component of the study, we used a qualitative description design [22] to understand what factors are related to the acceptance and usability of the FEPSim device. Qualitative description is appropriate when seeking to provide a descriptive summary of the experiences and opinions of a group of people in relation to a phenomenon [23,24].

### Setting

This study was conducted in 2 health care facilities located in Edmonton, Alberta, Canada, namely the Royal Alexandra Hospital Outpatient Clinic and Glenrose Rehabilitation Hospital Specialized Rehabilitation Outpatient Program Hand Class.

### Participants, Sample Size, and Recruitment

A purposive sampling method was used in this study. Hand therapists from hand therapy services that used the FEPSim device were recruited, as they could provide insight into what factors have an influence on the acceptance and usability of the FEPSim device. To ensure we reflected the diversity of experiences appropriately, we intentionally recruited hand therapists from the 2 clinical sites. All the participants were required to have used the FEPSim device. A total of 10 interviews were conducted, 1 with each participant. Hand therapists did not receive any incentive for participating in this study.

### Ethical Considerations

Ethics approval was obtained from the University of Alberta Research Ethics Board and in accordance with the Declaration of Helsinki (study protocol and approval number: Pro00095587). Written informed consent was obtained from the participants prior to their participation in this study [25]. This study is part of a larger study registered at the International Registered Report Identifier (IRRID).

### Data Collection Procedures

We used semistructured interviews to ensure that we collected a broad range of perspectives [22,26] regarding the technology acceptance and usability of the device. The semistructured interviews had 16 questions. During the semistructured interviews, hand therapists responded to questions such as the following: Was the FEPSim useful? Was learning to use the FEPSim easy? Was using the FEPSim well-suited to your needs? Do people who are important to you think that you should use the FEPSim? Do you plan to use the FEPSim in the near future?

They also described actual use of the FEPSim, if applicable. The semistructured interviews allowed the respondents to express themselves in their own manner and pace [27]. The interviews were conducted face-to-face.

The project coordinator (YL) conducted 10 face-to-face semistructured interviews over the course of 14 months. Each interview began with the project coordinator presenting the study’s background and purpose. The interviews were completed in a range from a minimum of 15 minutes to a maximum of 45 minutes.

The semistructured interview questions (topic-guided) examined the technology acceptance and usability of the FEPSim device. The semistructured interview questions were developed, and their face validity was determined by obtaining feedback from 2 co-authors (AMC and AMRR) who had expertise in usability and technology acceptance research [28,29].

To verify the preliminary results with the participants [30], the participants were asked to read their interview transcripts for consistency. This was done to allow the participants to correct any misunderstandings, to further expand on their ideas, and to add comments regarding the technology acceptance of the FEPSim device if any had been missed.

### Data Analysis

The semistructured interviews were audio recorded and transcribed verbatim by a professional transcription service. The transcripts were read and reviewed multiple times to ensure accuracy [31]. Content analysis [32] guided our data analysis. The transcripts were annotated and coded based on their content. The codes were then organized into subcategories. After analyzing each semistructured interview, we compared the findings between the different participants. A conceptually clustered matrix was used to compare and contrast the responses. The data from each semistructured interview were summarized in a table and cross referenced. In order to achieve saturation, we used a data saturation model (ie, relates to the degree to which new data repeat what was expressed in previous data). Microsoft Excel software was used to conduct the data analysis.

In this study, we adopted verification strategies such as methodological coherence, sampling adequacy, concurrent data collection and analysis, and theoretical thinking in order to be more rigorous during the data collection and analysis [33]. We also adopted the verification strategies proposed by Morse et al [33] to enhance rigor during data collection and analysis. We supplemented these with aspects of trustworthiness strategies such as verifying data accuracy, peer debriefing, and keeping an audit trail [34].

## Results

### Participants

Table 1 shows the participants’ demographics. The sample comprised 10 therapists from the Royal Alexandra Hospital Outpatient Clinic (n=5) and the Glenrose Rehabilitation Hospital Specialized Rehabilitation Outpatient Program Hand Class (n=5).

**Table 1:** Participant characteristics (n=10).

Variable			Therapists’ results
Age (years), mean (SD)			40.20 (6.63)
Years providing hand therapy, mean (SD)			13.10 (6.95)
**Sex, n (%)**			
	Male	0 (0)	
	Female	10 (100)	
**Background education, n (%)**			
	Occupational therapy	7 (70)	
	Physical therapy	0 (0)	
	Other	3 (30)	
**Highest level of education, n (%)**			
	Bachelor’s degree	6 (60)	
	Master’s degree	2 (20)	
	Diploma	2 (20)	

### Factors Affecting the Technology Acceptance and Usability of the FEPSim Device

**Figure 1:**
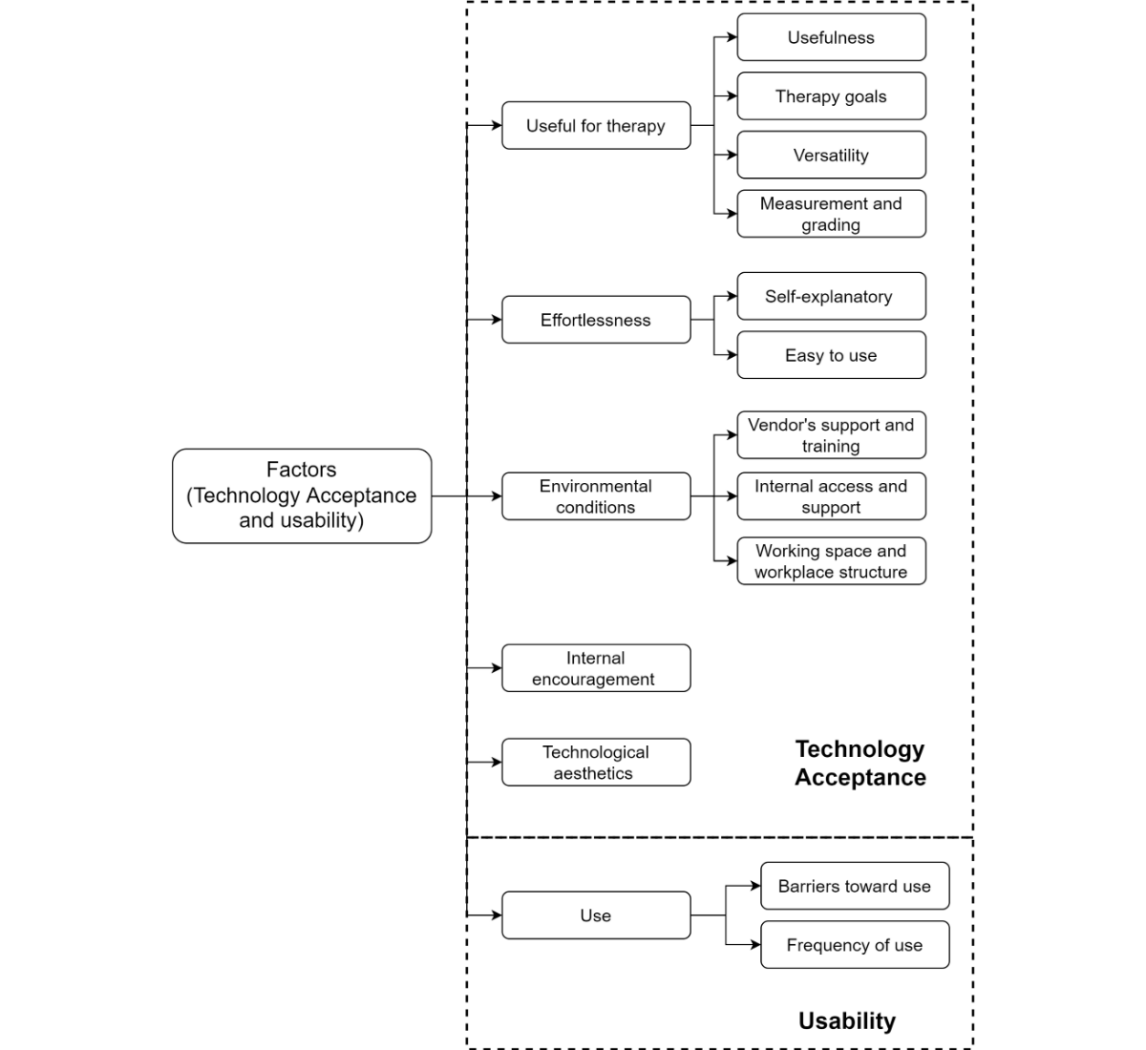
Factors that affects acceptance and usability of the FEPSim device.

The data generated 6 categories (hereafter, factors) as being critical aspects of the acceptance and usability of the FEPSim device. These factors were useful for therapy, effortlessness, environmental conditions, internal encouragement, technological aesthetics, and use. Each factor was further divided into subcategories as described in the following sections (see Figure 1 for more details).

#### Useful for Therapy

One aspect of the device that was useful for therapy was described by the therapists as how its features facilitated therapy sessions with patients and helped to achieve their therapeutic goals. Subcategories of this included usefulness, therapy goals, versatility, and measurement and grading.

Usefulness is related to how the features of the FEPSim device can be used in combination with other strategies and modalities to facilitate the provision of hand therapy to patients who have a lower-level arm injury or disorder and who have moderate to high functioning. The participants highlighted that using the FEPSim device may provide patients with independence during therapy, as the therapist can teach them how to change tools independently or the patients will know what repetitions they have done of a given exercise, which will facilitate the provision of therapy to groups (eg, hand classes). The participants also found it useful that the FEPSim device allows daily activities to be simulated.

Like, the number is there in front of people. So, they can see how many times they’ve turned the tool. And the ways that you adjust the resistance on it is more precise, and so I think it’s more effective and more professional than the older tool.

P8

I find it’s useful because a lot of the tools on it are—simulate, kind of, every day functional things they do, like the doorknob and the twisting.

P7

Therapy goals were described in terms of how the FEPSim device helps therapists work on patients’ strength and endurance building, range of motion, grasp and release or grip and pitch, and functional goals. However, one participant commented that the device is not useful for manipulation goals.

I think I used it if I wanted to get a certain work on a certain movement pattern. So, if there was, you know, the knob or the lever or the key grasp or, you know, any of the other sort of grasps that I felt like, you know, I wanted to target, it was good. It was good in that respect... Yes. I felt like, like I said, I used it to achieve certain movement patterns and for getting in the strength and endurance that I needed.

P9

I don’t tend to use it for manipulation goals, because you’re not really manipulating, you’re grabbing and releasing or pinching and releasing, you’re not having to pick up and manipulate stuff within your fingertips, so.

P4

Versatility is related to specific features that make the FEPSim device a single tool that provides many different options and attachments that can be used for several therapeutic goals with patients. For example, the device can be adjusted for resistance, it has several attachments that allow different grasp and grip patterns to be worked on, it counts the number of repetitions, and it is slick. The participants also used descriptors such as the FEPSim is an “all-in-one device,” thus highlighting that neither the patients nor the therapists have to walk around to use different tools, but rather, the FEPSim provides everything they need in one device.

I think the adjustability is good in terms of, you know, being able to use one thing for many usages.

P9

I like the versatility with all the different, like, attachments and heads that you can use. And the fact that you can have a great range of adjustability for resistance.

P8

It was useful because then I could track and I could plan to make increases and to increase the program and the resistance to reach goals of, you know, increased strength and that type of thing.

P7

Measurement and grading are related to the device’s features that allow different aspects of the treatment to be counted, graded, measured, and monitored over time. The participants highlighted as positive features that the FEPSim counts the repetitions of the exercises and allows the therapist to grade the resistance, the amount of rotation, and the difficulty of different grasps and grips using the real-life attachments; thus, the therapist controls the strength, range of motion, and hand patterns the patient needs to use during a session. All of these features allow the therapists to use objective data and provide feedback to the patients, which helps with the transferability of skills gained from the device to the use of real-world objects.

What I really like is the counter. So, the patients get direct feedback. They don’t have to rely on counting the repetitions. For traditional pieces of equipment, they don’t have that visual feedback. So, I like that. As they’re doing it, they can see the counter, so I can say stop at 20.

P10

I like that it counts the reps and that you can increase the strength.

P1

#### Effortlessness

Effortlessness within the context of this study was defined as how easy it is to learn to use the device. The participants identified that the FEPSim device was self-explanatory and easy to use.

The self-explanatory aspect was related to how easy it was to learn or figure out how to use the device. The participants highlighted that learning how to use the device was easy and self-explanatory and that they were able to figure out on their own how to use it by asking their colleagues a few questions.

Yeah. Learning is pretty straightforward. Like, you look at it, and it’s fairly intuitive in how you set it up. Like, you pull the pin. You can see how the attachments only attach in a certain way. Yeah. And then the device got improved when you could do the—when you could push down the suction to get it off [to remove it from the table], because before the suction used to be really hard to get off.

P10

The easy-to-use aspect includes how easy it is for the device to be used, cleaned, set up, and transported, as well as how easy it is to teach patients how to use it. In general, most of participants commented that the FEPSim device is easy to use.

I don’t think setting it up takes a lot of effort. Sometimes, like, taking it off the table as I just described is a little bit more effortful.

P8

It’s pretty straightforward to use, pretty easy to clean.

P4

It’s useful in that it’s easy to transport and just have it sit at one station, and they can do a number of things without having to move around the room.

P7

#### Environmental Conditions

Environmental conditions were described in terms of how supportive the environment is with regard to using the FEPSim device. The participants commented on the support and training provided by the FEPSim device’s vendor and the institutional access and support.

The participants identified the vendor's support and training as being an important element for using the device’s features in full. The participants felt that they had not had formal training or direct contact with the vendor. One participant commented on having only a little in-service. It seems that formal training on how to use the device was not provided by the vendor, so the therapists had diverse experiences with their training. Discrepancies in training might affect how much the therapists used all the device’s features.

But I thought that it was—like, I was able to figure it out. Maybe I didn’t figure out everything, but I thought I got it to do what I wanted... Maybe I didn’t know that you could do it (supination and pronation) with that machine.

P9

I feel like the majority of the training that happens with the tool is just kind of from staff member to staff member.

P8

I think it would be a good idea if, moving forward, that there is a legend or an exercise sheet or—that shows different ways of using the tools in order to target different things. Because as it stands, I think everything was left just to me to try and put things together in order to reach the goals that I wanted.

P7

Institutional access and support are related to how much support from the hospitals was provided to use the FEPSim device and how available the device was for use during therapeutic sessions. The participants felt, in general, that the hospitals supported the use of the FEPSim device. The therapists commented that the device was available in general, although some barriers were identified regarding its complete accessibility such as the FEPSim sometimes being located in an intervention room different from the one used to provide treatment. The participants also commented that they learned how to use the device mainly from their colleagues and that they did not have direct contact with the vendor when they had an issue with the device. The contact was through technology leaders who are therapists whose duties include promoting technology adoption at one of the hospitals.

Oh, see I’ve never dealt with [the vendor], it was always our technology leader that kind of would go to it, and I’ve never had a problem that I had to problem-solve with, but then it was kind of self-evident how to work the FEPS, so.

P4

I guess it’d be whether or not we’re treating in our room that has the FEPS. Because right now, we have two [FEPSim] in SROP [the outpatient service], but we have treatment spaces all over the hospital. So, if there’s no FEPS there, then I wouldn’t—or I couldn’t book the area with the FEPS and that would be a barrier.

P10

#### Internal Encouragement

Internal encouragement is related to the attitudes of people in the therapists’ human environment toward them using the FEPSim device as part of their treatment. The participants commented on feeling encouraged by their managers, supervisors, and colleagues to use the FEPSim device but that they were able to make the decision about whether to use it or not with their patients.

They’re good at encouraging us to and providing the education and then allowing us to have that therapeutic decision making if we use it or not.

P5

They [managers, supervisors, colleagues] encourage it [the FEPSim device], like the therapists do the assessments and they’ll add that on the treatment plan, or we can add it too, and that happens regularly.

P1

#### Technological Aesthetics

Technological aesthetics was described in terms of how the FEPSim device’s appearance contributed to its acceptance. The participants commented on how cool, innovative, and high-tech the device looked, which motivated the patients to engage during sessions when it was used.

I mean, the FEPS looks good. It does. It looks pretty sleek. And I think patients get impressed by the look. Actually, my patient really wanted to—the one that was in the control—wanted to use it. So, I think that might be a motivating factor for patients.

P9

...if I talk about like how it’s made, people are always, think it’s really cool, because it’s on a 3D printer. So, the patients get excited about it, because they just think that it’s cool and they like the way it looks. [laugh] Yeah.

P10

#### Use

Use, in the context of this study, is related to the factors that influence the actual use of the device during therapeutic sessions. The subcategories identified by the participants are related to the barriers regarding use and frequency of use.

The barriers against the actual use of the FEPSim device included (1) COVID-19–related restrictions, (2) patient conditions, and (3) therapeutic goals that were clearly focused on functional outcomes. The participants commented on how the COVID-19 pandemic had been having a huge impact on the use of the device, as the measures to control the spread of the virus changed the frequency of the therapeutic sessions and the mode of delivery (eg, from in-person to telerehab). The therapists commented that, if the device was available at home, the patients could use it on a regular basis. However, as this was not the case, during the sessions at the hospital, the therapists opted to teach activities and exercises that the patients could practice with the resources they had at home. Lack of active movement of the wrist, lack of grip, pain, and cognitive issues were identified as factors related to the patients’ conditions that limited the therapists from incorporating the FEPSim device during the treatment sessions. One participant commented that the FEPSim was “abstract” for patients with cognitive issues whose sessions needed to focus on actual functional tasks (eg, getting dressed or doing up their zipper), which are not possible with the device. Having patients with cognitive issues was an example of how the device was not appropriate for use with cases for whom the therapeutic goals needed to be strongly focused on functional, real-world outcomes.

Well, because COVID, I don’t know what my near future looks like, but right now I’ve been doing a lot in outpatients, right now my whole caseload is on Zoom.

P5

Frequency of use is related to how frequently the therapists used the FEPSim device. The participants commented on how they used the device fairly frequently but not in every single session with the patients. Frequency of use depended on the patients’ needs, the availability of the device, and how the session turned out.

I think it’s been used fairly frequently but not on everybody—depending on the patient.

P10

## Discussion

### Principal Findings

In this study, we aimed to understand what factors affect the technology acceptance and usability of the FEPSim device for hand therapy by therapists at 2 hospitals in Alberta, Canada. In doing so, by using a qualitative description design, we analyzed data from 10 semistructured interviews conducted with hand therapists that used the FEPSim. Overall, 6 factors were found to be critical aspects of the acceptance and usability of the FEPSim device. These factors were useful for therapy, effortlessness, environmental conditions, internal encouragement, technological aesthetics, and use.

Our findings revealed that the FEPSim device was useful for hand therapy. The usefulness of the FEPSim device lies in the fact that it is a versatile tool with a variety of measurement and grading systems that allow therapists to tailor their treatment plans to achieve their clients’ hand therapy goals. This finding is consistent with the results of previous studies about technology acceptance and usability in mobile health, health, and rehabilitation and assistive technologies [28,35-37]. For example, the study by Liu et al [35] aimed to explore the technology acceptance and usability of GPS technology among persons living with dementia and family caregivers; they found that usefulness had a significant influence on the acceptance of GPS. In the study, GPS technology provided peace of mind for caregivers and more independence for people living with dementia [35]. In another study, Liu et al [28] reported that usefulness was the most significant factor in the acceptance of new rehabilitation technologies. In other words, how rehabilitation technologies can help therapists to achieve their therapeutic goals with clients was the most important factor in determining therapists’ acceptance and use of these new rehabilitation technologies [28].

It is important to note that the versatility of the FEPSim is added value for the device that allows therapy to be achieved. In other words, the therapists acknowledged that the FEPSim device has a relative advantage over traditional devices (including old FEPS models [ie, wood-based devices]) that therapists had been using. Relative advantage is understood to be the degree to which using the FEPSim device is perceived as being better than using its precursor or other devices [38]. The relative advantage of the FEPSim accounts for a device that is more usable and functional for therapy. This finding reassures rehabilitation technology designers that they should hear the voices of therapists before embarking on designing and creating these technologies. In other words, the use and implementation of co-design and co-creation strategies should be paramount for rehabilitation technology designers.

We found that using the FEPSim device was effortless. The FEPSim device is self-explanatory and easy to use not only for therapists but also for patients. By having a device that is self-explanatory, therapists meant that the FEPSim is very intuitive to use and that it is easy to remember how to use it. Simply put, the FEPSim device is memorable. The scholarly literature defines memorability as a feature of a technology that is easy to remember, so that the casual user is able to return to use the technology after some period of not having used it, without having to learn everything all over again [39]. On the other hand, ease of use is understood to be the degree to which a person believes that using a technology would be free of effort [38]. The scholarly literature in the field of technology acceptance and usability of mobile applications for health, health, and rehabilitation and assistive technologies shows that ease of use is becoming a less important factor with regard to technology acceptance as long as the technology shows signs of usefulness in achieving therapeutic goals. For example, one study found that therapists were not influenced by the degree of difficulty of using new rehabilitation technologies [28]. The same is true for another study that assessed the technology acceptance of a mobile application that was intended to support the workflow of health care aides who provided services to older adults residing in a care facility [37]. More surprisingly, a further study found that the ease of use of a GPS technology mattered inversely to its technology acceptance. In other words, contrary to what many technology acceptance theories predict (eg, [38]), while the users perceived that the GPS was hard to use, they would still have continued to use the device if they were able to do so [35]. Since our study was a qualitative study, it was impossible for us to determine how important effortless was in comparison to the other factors. Therefore, it would be worth exploring through a quantitative study whether the FEPSim’s ease of use is a salient factor with regard to technological acceptance and the specific weight of this factor compared with usefulness, for example.

The therapists believed that they had created all the conditions to use the FEPSim device. Environmental conditions included the vendors’ support and training, internal access to the device and support, and working space and workplace structure. Environmental conditions, also known as facilitating conditions in some technology adoption theories such as the Unified Theory of Acceptance and Use of Technology (UTAUT), are defined as the degree to which an individual believes that all the conditions exist to support the use of a technology [38]. This result did not surprise us, as there is an extensive body of literature in the field of technology acceptance and use that points toward facilitating conditions as a salient factor in technology acceptance and use [40].

The aesthetics of the FEPSim device motivated its use. In other words, the patients liked the way the FEPSim device looks and the way the device was made (eg, 3D-printed). This finding is consistent with the scholarly literature and, at the same time, is interesting because it has some implications. For example, a recent study aimed to identify what factors are related to the acceptance and usability of locator devices that are important to individuals with dementia and other stakeholders. The study found that aesthetic appeal was a factor in technology acceptance [29]. The implication of our finding lies in the fact that aesthetic appeal might have been a motivating factor for the patients to use the device, which in turn might have influenced the therapists’ decision to adopt the FEPSim technology. In other words, the clients drove the therapists’ decision to adopt the technology. Motivation, which is more formally called hedonic motivation in some technology acceptance theories (eg, UTAUT2) and which is defined as the fun or pleasure derived from using a technology [41], has been shown to be an important determinant in technology acceptance and use [42]. Other scholars have found similar results regarding “positive feedback” that influenced mutual technology acceptance between dyads of users. For example, in one study, caregivers and clients living with dementia encouraged each other to use GPS devices in order to address the burden on caregivers that is associated with anxiety about their clients getting lost [35]. From a research perspective, it would be worth studying the phenomenon of technological acceptance further, following a dyadic approach rather than exploring individual perspectives and beliefs.

### Limitations

This study has one important limitation. The data were collected from participants who were therapists from 2 health care institutions in the public sector only. This limited the potential for generalizing the results, for example, to therapists from other provinces or the private health care sector in Canada or in other countries. Regardless of this limitation, the findings can be used to understand the technology acceptance and usability of the FEPSim device. They also serve as a starting point for future research, specifically quantitative and mixed methods research.

### Conclusions

Overall, our findings suggest that the factors useful for therapy, namely effortlessness, environmental conditions, internal encouragement, technological aesthetics, and use, affected the technology acceptance and usability of the FEPSim device for hand therapy by therapists. In conclusion, the FEPSim device was widely accepted by therapists. The use of the FEPSim device is a feasible alternative for supporting hand therapy.

## Abbreviations

AICE: Accelerating Innovation Into Care
IRRID: International Registered Report Identifier
UTAUT: Unified Theory of Acceptance and Use of Technology
